# Plasma Cytokine Atlas Reveals the Importance of TH2 Polarization and Interferons in Predicting COVID-19 Severity and Survival

**DOI:** 10.3389/fimmu.2022.842150

**Published:** 2022-03-21

**Authors:** Lara Gibellini, Sara De Biasi, Marianna Meschiari, Licia Gozzi, Annamaria Paolini, Rebecca Borella, Marco Mattioli, Domenico Lo Tartaro, Lucia Fidanza, Anita Neroni, Stefano Busani, Massimo Girardis, Giovanni Guaraldi, Cristina Mussini, Alessandro Cozzi-Lepri, Andrea Cossarizza

**Affiliations:** ^1^ Department of Medical and Surgical Sciences for Children and Adults, University of Modena and Reggio Emilia School of Medicine, Modena, Italy; ^2^ Infectious Diseases Clinics, Azienda Ospedaliera-Universitaria (AOU) Policlinico and University of Modena and Reggio Emilia, Modena, Italy; ^3^ Department of Anesthesia and Intensive Care, Azienda Ospedaliera-Universitaria (AOU) Policlinico and University of Modena and Reggio Emilia, Modena, Italy; ^4^ Centre for Clinical Research, Epidemiology, Modelling and Evaluation, Institute for Global Health, London, United Kingdom; ^5^ National Institute for Cardiovascular Research, Bologna, Italy

**Keywords:** SARS-CoV-2, COVID-19, cytokines, chemokines, survival

## Abstract

Although it is now widely accepted that host inflammatory response contributes to COVID-19 immunopathogenesis, the pathways and mechanisms driving disease severity and clinical outcome remain poorly understood. In the effort to identify key soluble mediators that characterize life-threatening COVID-19, we quantified 62 cytokines, chemokines and other factors involved in inflammation and immunity in plasma samples, collected at hospital admission, from 80 hospitalized patients with severe COVID-19 disease who were stratified on the basis of clinical outcome (mechanical ventilation or death by day 28). Our data confirm that age, as well as neutrophilia, lymphocytopenia, procalcitonin, D-dimer and lactate dehydrogenase are strongly associated with the risk of fatal COVID-19. In addition, we found that cytokines related to TH2 regulations (IL-4, IL-13, IL-33), cell metabolism (lep, lep-R) and interferons (IFNα, IFNβ, IFNγ) were also predictive of life-threatening COVID-19.

## Introduction

The clinical phenotypes of severe acute respiratory syndrome type 2 (SARS-CoV-2) infection spans from asymptomatic or paucisymptomatic infection to critical or lethal Coronavirus disease 2019 (COVID-19). Epidemiological studies showed that being male or being elderly and certain medical conditions and co-morbidities, including obesity and hypertension, are risk factors for life-threatening COVID-19 (1, 2). However, even considering these factors, there is still a huge variability in the clinical outcome across infected individuals.

Multiple studies suggest that uncontrolled inflammation contributes to disease severity (3–5). Indeed, high levels of inflammatory markers, including D-dimer, which is a product of fibrin degradation, and lactate dehydrogenase have been observed in patients with severe disease (6). As a further proof that dysregulated inflammation contributes to disease severity, plasma levels of anti-inflammatory cytokines such as interleukin (IL)-1RA, IL- 10 and IL-19, were increased in pregnant women with asymptomatic or paucisymptomatic SARS-CoV-2 infection (7).

Nonetheless, in severe disease the levels of erythrocyte sedimentation rate (ESR) and C-reactive protein (CRP) were increased regardless of disease severity or presence of comorbidities (6). Procalcitonin was elevated in severe and critical patients. Neutrophilia was present in patients who progressed to acute respiratory distress syndrome (ARDS), and total lymphocyte count, and CD4+ T cells decreased in severe or critical patients (6). In addition, reduced monocyte counts, reduced T-cell functionality, monocyte dysfunctions, increased neutrophil-to-lymphocyte ratio have been described as important features of severe and critical COVID-19 (5, 8–10). However, routine clinical data are not yet sufficient to completely distinguish among COVID-19 severities and among COVID-19 and influenza or other similar respiratory diseases (4).

Alterations in plasma concentration of multiple cytokines, *i.e.* those released during the so-called cytokine storm, or hypercytokinemia, heavily contributes to disease progression (9–15). It has been shown that patients who did not survive presented significantly higher levels of interleukin (IL)-15 than those who recovered (16). Granulocyte-macrophage colony stimulating factor (GM-CSF) and IL-1α allowed to distinguish fatal COVID-19 from fatal influenza (4).

Since measuring an extended number of cytokines represents the best strategy to investigate complex pathologic conditions (17), the aim of our study was to evaluate the prognostic value of an extensive set of 62 plasma biomarkers measured at hospital admission to predict the risk of mechanical ventilation or death by day 28.

## Methods

### Study Design

This is a single-centre study, approved by the local Ethical Committee (Comitato Etico dell’Area Vasta Emilia Nord, protocol number 177/2020, March 11th, 2020) and by the University Hospital Committee (Direzione Sanitaria dell’Azienda Ospedaliero-Universitaria di Modena, protocol number 7531, March 11th, 2020). Eighty patients with severe or critical COVID-19 admitted at the Infectious Disease Clinics or Intensive Care Unit at University Hospital in Modena (Italy) in March-May 2020 were included in this study. Each participant provided informed consent according to Helsinki Declaration, and all uses of human material have been approved by the same Committees.

### Blood Collection

Blood samples (up to 20 mL) were obtained, at hospital admission, after informed consent. Plasma was collected, centrifuged at 800 rpm for 20 minutes and stored at -80°C until use.

### Quantification of Cytokine Plasma Levels

The plasma levels of 62 molecular species were quantified by using the Luminex platform (Human Cytokine Discovery, R&D System, Minneapolis, MN) and the following kits: Human XL Cytokine Luminex Performance Panel Premixed Kit (cat.no FCSTM18), Human Luminex Discovery Assay (cat.no LXSAHM-13), Human Luminex Discovery Assay (cat.no LXSAHM-05), all manufactured and distributed by R&D systems. The following molecules were quantified: APRIL, B-cell activating factor (BAFF), bone morphogenetic protein (BMP) 2, BMP4, BMP7, CD40L, C-C Motif Chemokine Ligand (CCL) 2, CCL3, CCL4, CCL5, CCL11, CCL19, CCL20, C-X-C Motif Chemokine Ligand (CXCL) 1, CXCL2, CXCL10, CX3CL1, epidermal growth factor (EGF), fibroblast growth factor basic (FGF basic), granulocyte-colony stimulating factor (G-CSF), granulocyte-macrophage colony stimulating factor (GM-CSF), interferon (IFN)-α, IFN-β, IFN-γ, IL-1α, IL-1 receptor antagonist (IL-1RA), IL-1β, IL-2, IL-3, IL-4, IL-5, IL-6, IL-6RA, IL-7, IL-10, IL-11, IL-12p70, IL-13, IL-15, IL-17, IL-17C, IL-17E, IL-18, IL-19, IL-23, IL-27, IL-33, FAS, FASL, FLT-3 ligand, granzyme B (GRZB), leptin (lep), leptin R (lep-R), osteopontin (OPN), programmed death ligand-1 (PD-L1), platelet derived growth factor (PDGF)-AA, PDGF-AB/BB, transforming growth factor (TGF)-α, transmembrane activator and CAML interactor (TACI), tumour necrosis factor (TNF), TNF-related apoptosis-inducing ligand (TRAIL), vascular endothelial growth factor (VEGF), according to the manufacturer’s instruction. Molecules were classified into 12 functional groups as follows: anti-inflammatory response (IL-10, IL-19, IL-1RA, IL-6RA), apoptosis (FASL, FAS, PD-L1), B cell regulation (APRIL, BAFF, IL-5, TACI, CD40L), cell metabolism (lep, lep-R), chemotaxis/activation/recruitment (CCL2, CCL3, CCL4, CCL5, CCL11, CCL19, CCL20, CXCL1, CXCL2, CXCL10, CX3CL1, OPN), cytotoxicity (GRZB, TNF, TRAIL), T cell differentiation (IL-12p70, IL-15, IL-27, IL-2, IL-7), growth factors (EGF, FGF basic, FLT-3 ligand, GM-CSF, G-CSF, IL-11, IL-3, PDGF-AA, PDGF-AB/BB, VEGF), inflammation (IL-17C, IL-17E, IL-17, IL-18, IL-1β, IL-1α, IL-23, IL-6), interferons (IFN-α, IFN-β, IFN-γ), morphogenetic factors (BMP2, BMP4, BMP7, TGF-α) and TH2 regulation (IL-4, IL-13, IL-33).

While acknowledging that there is considerable overlap in cascading immunological relationships, and downstream effects among many of these biomarkers, for ease of comparison the 62 markers are displayed by broadly grouping them into 12 functional categories, that were defined as previously indicated.

### Statistical Analysis

The primary outcome was the clinical severity binary endpoint of experiencing invasive mechanical ventilation (IMV) or death during hospitalization (cases) *vs.* hospital discharge (controls) by day 28 from hospital admission. Main demographic characteristics were compared between cases and controls by means of chi-square test (for categorical variables) and Mann-Whitney test (for numeric variables). The association of each of the 62 biomarkers measured at hospital admission and clinical severity was assessed in separate comparisons of biomarker levels between cases and controls; an advantage of such prospective comparisons is that the temporality of the biomarker level and disease severity is known (i.e., an elevated value for the biomarker was observed before and not after the progression of the disease). Historically, temporality together with a plausible hypothesized biological mechanism has helped to establish causal links when trials could not be performed (e.g. smoking and risk of lung cancer). To reduce the impact of outliers and to account for the positively skewed distribution of the biomarkers, values were categorized in tertiles and log_10_ transformed. Data were shown using dot-plots indicating median and IQR. Median levels of biomarkers between cases and controls were compared using the Mann-Whitney test. A logistic regression was used to evaluate the association of each biomarker with the clinical severity outcome. Odds ratios (ORs) for the upper tertile versus the lowest tertile are cited along with 95% confidence intervals (CIs) and *p*-values. Analyses that used the log_10_ transformed biomarkers as a continuous covariate were also carried out and ORs per 1 log_10_ difference in the biomarker shown; multivariable estimates were adjusted for gender, age and extent of co-morbidity (age-unadjusted CCI). To determine the relationship of multiple biomarkers with clinical severity, we took advantage of the functional groupings listed in the previous paragraph. A global non-parametric test procedure proposed by O’Brien for multiple endpoints was used (18). With this approach, each marker in the raw scale within each of the functional grouping is ranked from lowest to highest, the ranks of the individual markers are summed for each patient. We refer to the sum of the ranks as the “biomarker score”. This biomarker score is then compared for patients who experienced IMV/death vs. those who were discharged with logistic regression models as described above. Advantages of this procedure are simplicity and increased power if the biomarkers within a category all trend in the same direction. A disadvantage is that while the global test identifies biomarker groupings that are significant, it does not provide information on which markers are driving the statistical significance. However, the information of the importance of single markers were additionally obtained by the likelihood ratio test that tested the significance of adding all the markers (in the log_10_ scale) in a functional category to a base model that only included gender, age and age-unadjusted CCI. Statistical analyses were performed using SAS (Version 9.4, Carey NC, USA).

## Results

Eighty patients with severe or critical COVID-19 admitted at the Infectious Disease Clinics or Intensive Care Unit at University Hospital in Modena (Italy) in March-May 2020 were included in this study. Age, gender, race/ethnicity and comorbidities were all collected on admission. Several laboratory variables, such as blood cell count, alanine-aminotransferase (ALT), international normalized ratio (INR), creatinine, estimated glomerular filtration rate (eGFR), CRP, interleukin (IL)-6, procalcitonin, D-dimer, haemoglobin, lactate dehydrogenase were also measured at hospital entry.

By day 28, fifty-three patients were discharged after recovery whereas twenty-seven received mechanical ventilation and/or died during the observation period. Our data confirm that older age is a risk factor for mechanical ventilation and/or death, as these patients had a median age of 72 when compared to discharged patients who had a median age of 60 (P=0.008) (**Table 1**). There was no evidence for a difference in the prevalence of comorbidities at admission between cases and controls. These include obesity, ischemic cardiomyopathy, COPD, connective tissue disease, cerebro-vascular disease, mild liver disease, diabetes, chronic kidney failure, solid tumors, liver failure, haematologic diseases, peptic ulcer diseases, dementia, arterial hypertension, chronic heart failure, peripheral vascular disease (**Table 1**). This univariable analysis also showed that the proportion of neutrophils was higher in cases vs. controls (89.2% vs 74.6%; P<0.001) and patients who underwent mechanical ventilation and/or died had a lower number of lymphocytes (930.0 vs 1,891/mm^3^; P=0.045) when compared to discharged patients. Cases also had a higher level of procalcitonin (0.3 vs 0.1 ng/mL; P=0.004), D-dimer (2,165 vs 1,040 ng/mL; P=0.001), lactate dehydrogenase (762.0 vs 603.0 U/L; P=0.044). In contrast, there was no evidence for a difference in leukocyte count (p=0.234), platelet count (p=0.281) and creatinine concentration (p=0.1365) by groups (**Table 1**).

**Table 1:** Demographic, clinical characteristics and baseline laboratory parameters of patients involved in the case-control study.

Characteristic	Cases^#^	Controls	p-value^*^	Total
	N = 27	N = 53		N = 80
**Age, years**				
Median (IQR)	72 (67, 77)	60 (52, 74)	0.008	65 (55, 75)
**Gender, n(%)**				
Female	4 (14.8%)	15 (28.3%)	0.183	19 (23.8%)
**Ethnicity, n(%)**				
Caucasian	27 (100.0%)	50 (94.3%)	0.457	77 (96.3%)
Black	0 (0.0%)	2 (3.8%)		2 (2.5%)
Asian	0 (0.0%)	1 (1.9%)		1 (1.3%)
Ispanic	0 (0.0%)	0 (0.0%)		0 (0.0%)
**BMI**				
Median (IQR)	25.8 (24.6, 27.6)	29.0 (25.6, 33.6)		27.7 (25.1, 31.3)
**Comorbidities, n(%)**				
>=1	23 (85.2%)	37 (69.8%)	0.136	60 (75.0%)
Obesity	2 (12.5%)	18 (37.5%)	0.064	20 (31.3%)
Ischemic cardiomyopathy	14 (51.9%)	27 (50.9%)	0.939	41 (51.3%)
COPD	8 (29.6%)	18 (34.0%)	0.697	26 (32.5%)
Connective tissue disease	8 (29.6%)	15 (28.3%)	0.902	23 (28.8%)
Cerebro-vascular disease	8 (29.6%)	14 (26.4%)	0.762	22 (27.5%)
Mild Liver disease	1 (6.3%)	0 (0.0%)	0.128	1 (1.9%)
Diabetes	13 (48.1%)	25 (47.2%)	0.934	38 (47.5%)
Chronic kidney failure	10 (37.0%)	14 (26.4%)	0.330	24 (30.0%)
Solid tumour	10 (37.0%)	18 (34.0%)	0.786	28 (35.0%)
Liver failure	6 (22.2%)	12 (22.6%)	0.966	18 (22.5%)
Hematologic disease	1 (6.3%)	2 (5.4%)	0.904	3 (5.7%)
Peptic ulcer disease	1 (6.3%)	3 (8.1%)	0.816	4 (7.5%)
Dementia	6 (22.2%)	12 (22.6%)	0.966	18 (22.5%)
Arterial hypertension	15 (68.2%)	27 (64.3%)	0.757	42 (65.6%)
Chronic heart failure	4 (25.0%)	5 (13.5%)	0.311	9 (17.0%)
Peripheral vascular disease	5 (31.3%)	11 (29.7%)	0.913	16 (30.2%)
CCI, mean (SD)	6.3 (4.8)	5.4 (4.7)	0.363	5.7 (4.7)
**Main delays**				
Days from symptoms onset to hospitalisation, median (IQR)	7 (5, 8)	7 (4, 9)	0.845	7 (4, 9)
Days from symptoms onset to ICU, median (IQR)	10 (6, 12)	10 (8, 14)		10 (7, 12)
Days from hospitalisation to ICU, median (IQR)	2 (0, 3)	2 (0, 5)		2 (0, 4)
**Baseline laboratory parameters**				
Leukocytes, /mm^3^, Median (IQR)	6,840 (5,050; 12,420)	5,840 (5,140; 8,340)	0.234	6,305 (5,095; 8,435)
% neutrophils, Median (IQR)	89.2 (77.3, 91.7)	74.6 (60.2, 83.0)	<0.001	77.6 (65.5, 86.6)
Lymphocytes, Median (IQR)	930.0 (680.0; 2,028)	1,891 (1,050; 2,390)	0.045	1,661 (870.0; 2,347)
Platelets, 10^3^/mm^3^, Median (IQR)	181.0 (130.0, 283.0)	205.0 (181.0, 248.0)	0.281	199.0 (161.5, 254.5)
Alanine amino-transferase (ALT), U/L, Median (IQR)	45.0 (24.0, 62.0)	29.0 (21.0, 39.0)	0.036	33.0 (22.0, 57.5)
INR, Median (IQR)	1.2 (1.1, 1.2)	1.1 (1.0, 1.1)	0.007	1.1 (1.0, 1.2)
Creatinine, mg/dl, Median (IQR)	1.1 (0.5, 1.5)	0.9 (0.8, 1.1)	0.136	0.9 (0.8, 1.1)
eGFR, ml/min, Median (IQR)	74.2 (46.3, 91.3)	89.2 (74.5, 99.7)	0.014	86.1 (63.6, 98.3)
60+, ml/min, n(%)	17 (63)	46 (86.8)	0.044	63 (78.8)
31-60, ml/min, n(%)	6 (22.2)	5 (9.4)		11 (13.8)
0-30, ml/min, n(%)	4 (14.8)	2 (3.8)		6 (7.5)
C-reactive protein, mg/l, Median (IQR)	15.0 (6.0, 22.0)	12.0 (5.0, 17.0)	0.085	14.0 (5.5, 19.5)
IL-6, pg/ml, Mean (Range)	781.2 (6,920 – 5,785)	535.1 (4,140 – 11,863)	0.11	617.2 (4,140 – 11,858)
Procalcitonin, ng/ml, Median (IQR)	0.3 (0.2, 1.5)	0.1 (0.1, 0.3)	0.004	0.2 (0.1, 0.4)
D-dimer, ng/ml, Median (IQR)	2,165 (960.0; 4,110)	1,040 (650.0; 1,630)	0.001	1,170 (700.0; 2,120)
0-500 ng/ml, n(%)	1 (3.8)	7 (13.2)	0.007	8 (10.1)
501-4000 ng/ml, n(%)	18 (69.2)	44 (83.0)		62 (78.5)
>4000 ng/ml, n(%)	7 (26.9)	2 (3.8)		9 (11.4)
Haemoglobin, g/l	12.3 (11.2, 14.5)	13.0 (11.6, 14.1)	0.586	12.9 (11.5, 14.1)
Lactate dehydrogenase, U/l	762.0 (547.0; 1,022)	603.0 (508.0, 749.0)	0.044	633.5 (515.5, 825.5)

*Chi-square or Mann-Whitney test as appropriate. ^#^Mechanical ventilation or death.

**Figure 1:**
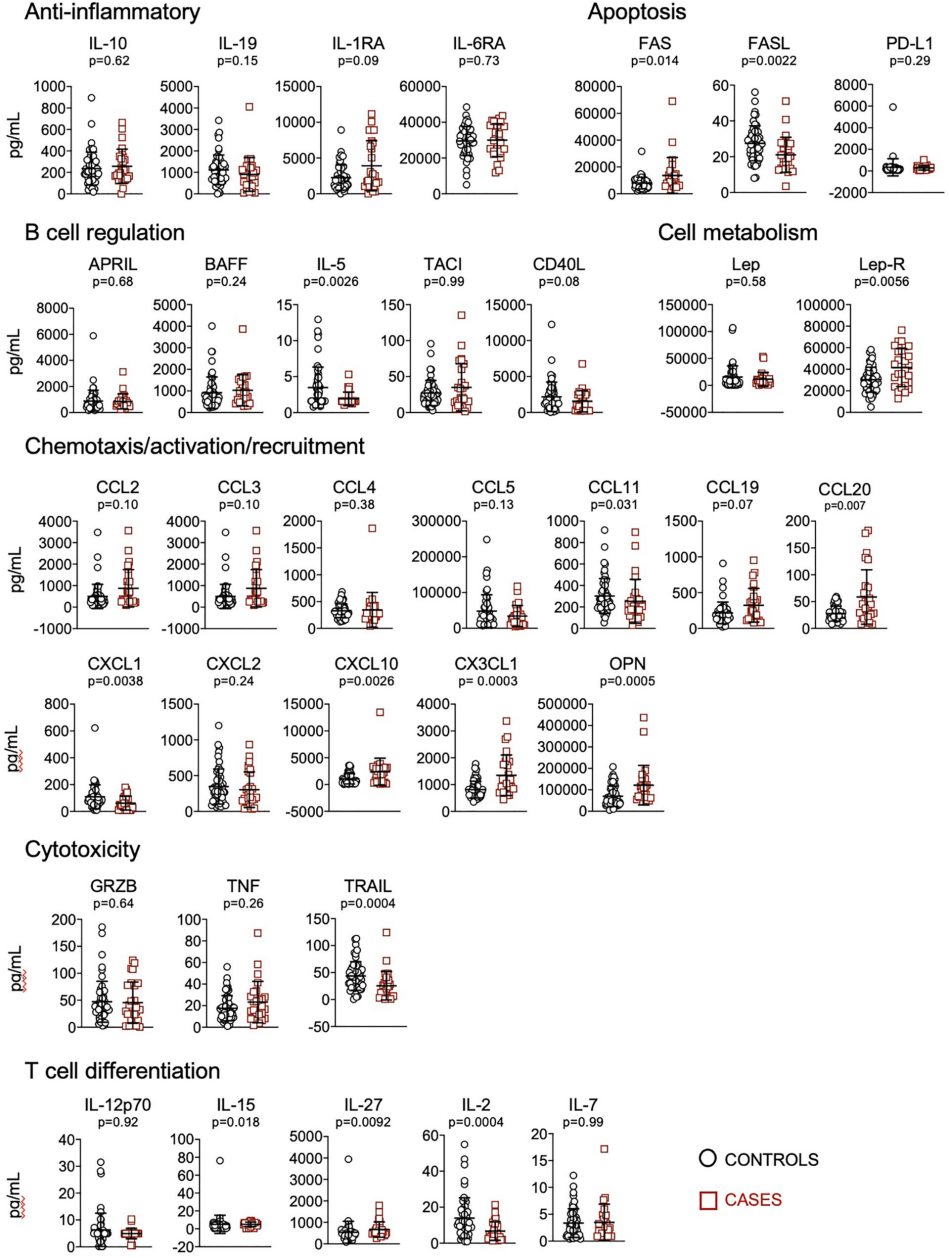
Quantification of cytokines and other biomarkers involved in anti-inflammatory responses, apoptosis, B cell regulation, cell metabolism, chemotaxis/activation/recruitment, cytotoxicity and T cell differentiation, in plasma obtained from controls (n=57) and cases (n=23). Data represent mean and standard deviation of the mean. Mann-Whitney test was used for statistical analysis.

**Figure 2:**
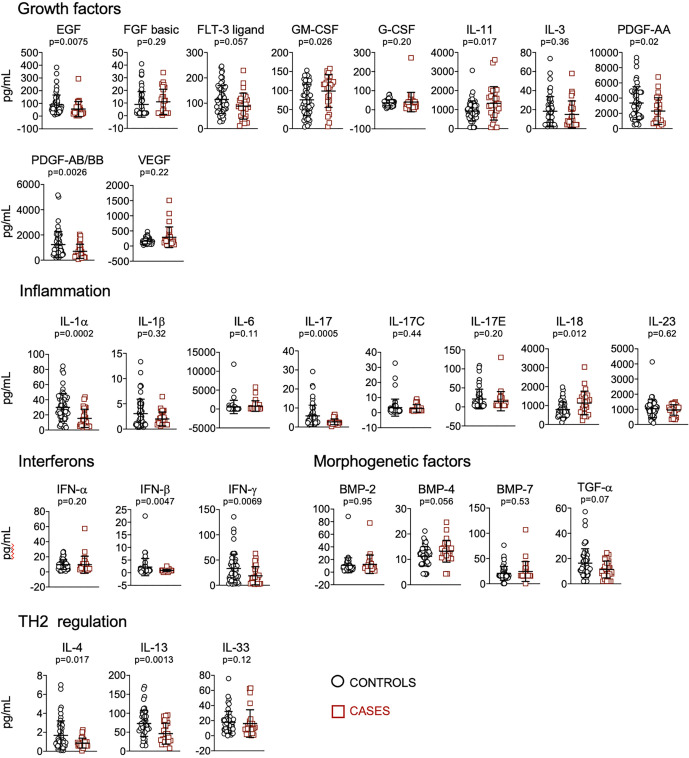
Quantification of cytokines and other biomarkers involved in growth factors, inflammation, interferons, morphogenesis, TH2 regulation, in plasma obtained from controls (n=57) and cases (n=23). Data represent mean and standard deviation of the mean. Mann-Whitney test was used for statistical analysis.

We quantified plasma levels of 62 cytokines, chemokines and other soluble factors involved in the regulation of the immune system. Considering single biomarkers, we found that patients who received mechanical ventilation or died presented significantly higher levels of FAS, lep-R, CCL20, CXCL10, CX3CL1, OPN, IL-27, GM-CSF, IL-11, IL-18, and decreased levels of FASL, IL-5, CCL11, CXCL1, TRAIL, IL-15, IL-2, EGF, PDGF-AA; PDGF-AB/BB, IL-1α, IL-17, IFN-β, IFN-γ, IL-4 and IL-13 (**Figures 1**, **2**).

**Figure 3:**
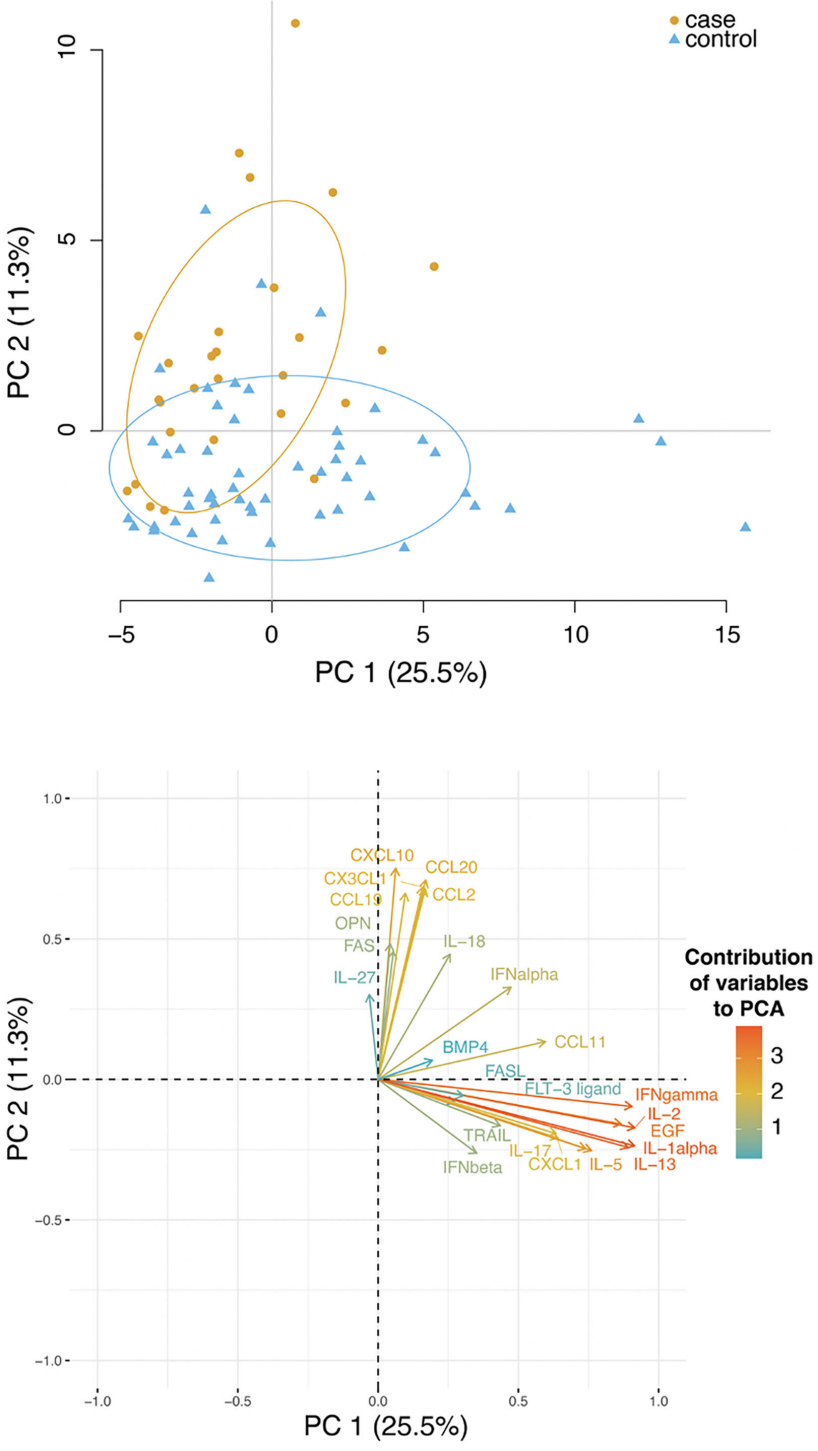
Plasma level of several cytokines and chemokines stratified controls and cases. Upper panel: principal-component analysis (PCA) analysis of cytokines, chemokines and other soluble molecules quantified across the cohort. Lower panel: a plot displays the variables as vectors, indicating the direction of each variable to the overall distribution. The strength of each variable is represented by colors: orange colour represents a strong contribution, light blue colour represents a milder contribution.

To get further insight, we performed a principal-component analysis (PCA) of cytokines/chemokines/soluble molecules across the cohort. We only considered two components as these already explained 36% of the total variability. The first principal component (PC1) was mostly responsible for the stratification of cases and controls (**Figure 3**, upper panel). A graph displaying the contribution of the different molecules on the principal components as arrows revealed that the most important contributors to PC1 and PC2 were the levels of CCL2, CCL19, CCL20, CXCL10, CX3CL1, IFNγ, IL-2, IL-13, IL-1α and EGF (**Figure 3**, lower panel). Notably, these molecules, that contribute to patients’ stratification in PCA, are also the main contributors their respective functional group.

Concerning the results of the logistic regression analysis, higher levels of FASL, FLT3 ligand, IFNβ, IL-1α, IL-5, IL-13, IL-17, TRAIL, EGF, CCL11, PDGF-AB/BB, IFNγ were associated with protection against mechanical ventilation or death, whereas higher levels of lep-R, CX3CL1, OPN, BMP-4, FAS, CCL2, CCL19, CCL20, IL-18, IL-27, CXCL10 were associated with higher risk of mechanical ventilation or death (**Table 2**).

**Table 2:** Odds ratio (OR) of invasive mechanical ventilation (IMV)/death from fitting a logistic regression model - log10 scale analysis.

Biomarker	Odds ratio	95% CI	p-value
**Anti-inflammation**			
IL-10	1.22	0.38, 3.94	0.744
IL-19	0.64	0.22, 1.85	0.407
IL-1RA	1.73	0.61, 4.93	0.303
IL-6RA	2.26	0.11, 44.51	0.592
**Apoptosis**			
FASL	0.03	0.00, 0.63	0.023
FAS	15.77	1.47, 169.6	0.023
PD-L1	1.68	0.36, 7.87	0.510
**B cell regulation**			
APR	2.03	0.30, 13.54	0.464
BAFF	3.29	0.52, 20.91	0.206
CD40L	0.62	0.19, 2.04	0.430
IL-5	0.02	0.00, 0.38	0.009
TACI	0.68	0.17, 2.78	0.590
**Cell metabolism**			
Lep-R	45.83	2.39, 879.1	0.011
Lep	1.10	0.33, 3.74	0.873
**Chemotaxis/activation/recruitment**			
CCL11	0.10	0.01, 0.84	0.034
CCL19	9.09	1.43, 57.74	0.019
CCL20	17.73	2.48, 126.8	0.004
CCL2	4.69	1.10, 20.07	0.037
CCL3	2.55	0.38, 17.14	0.336
CCL4	0.90	0.08, 9.97	0.930
CCL5	0.43	0.10, 1.76	0.240
CX3CL1	148.0	7.29, 3002	0.001
CXCL10	4.81	1.51, 15.34	0.008
CXCL1	0.09	0.02, 0.41	0.002
CXCL2	0.49	0.11, 2.09	0.334
OPN	30.52	3.89, 239.2	0.001
**Cytotoxicity**			
GRZB	0.70	0.25, 1.97	0.500
TNF	2.50	0.52, 11.98	0.253
TRAIL	0.20	0.05, 0.75	0.017
**T cell differentiation**			
IL-12p70	1.04	0.32, 3.41	0.951
IL-15	3.10	0.75, 12.79	0.118
IL-27	13.07	1.20, 142.8	0.035
IL-2	0.13	0.03, 0.58	0.008
IL-7	1.86	0.45, 7.69	0.390
**Growth factors**			
EGF	0.16	0.04, 0.70	0.015
FGF-basic	2.41	0.81, 7.14	0.113
FLT-3 ligand	0.06	0.01, 0.60	0.017
GM-CSF	5.30	0.94, 29.82	0.058
G-CSF	0.42	0.10, 1.88	0.258
IL-11	1.41	0.40, 4.94	0.594
IL-3	0.75	0.21, 2.69	0.663
PDGF-AA	0.24	0.05, 1.13	0.071
PDGF-AB	0.12	0.02, 0.61	0.010
VEGF	4.79	0.81, 28.26	0.083
**Inflammation**			
IL-17C	1.12	0.30, 4.20	0.869
IL-17E	0.66	0.21, 2.12	0.490
IL-17	0.04	0.00, 0.33	0.003
IL-18	16.28	1.55, 171.4	0.020
IL-1β	0.85	0.20, 3.57	0.825
IL-1α	0.09	0.02, 0.44	0.003
IL-23	0.64	0.08, 4.92	0.670
IL-6	2.08	0.95, 4.54	0.066
**Interferons**			
IFNβ	0.09	0.01, 0.73	0.025
IFNα	0.62	0.15, 2.60	0.513
IFNγ	0.29	0.09, 0.94	0.039
**Morphogenetic**			
BMP-2	1.46	0.27, 7.96	0.664
BMP-4	66.06	1.49, 2920	0.030
BMP-7	2.25	0.67, 7.53	0.189
TGFα	0.24	0.05, 1.28	0.095
**TH2 regulation**			
IL-13	0.04	0.00, 0.36	0.004
IL-33	0.85	0.37, 1.96	0.702
IL-4	0.32	0.08, 1.31	0.114

Adjusted for age, gender and age-unadjusted CCI.

When analysis was performed using the O’Brien method and the functional groupings described in the Methods, we found that groups related to TH2 regulations (IL-4, IL-13, IL-33), cell metabolism (lep, lep-R) and interferons (IFNα, IFNβ, IFNγ) were also predictive of life-threatening COVID-19. Specifically, patients who underwent mechanical ventilation or who died showed higher ranks of molecules involved in cell metabolism (p=0.0265), lower ranks for interferon response (p=0.0155) and lower ranks for TH2 regulation (p=0.0439) when compared to discharged patients (**Table 3**).

**Table 3:** Adjusted O’brien method analysis by groupings.

Group	Cases	Controls	Adj OR (95% CI)	p-value^$^	p-value^£^
	Median (IQR)	Median (IQR)			
Anti-inflammatory response ^a^	167 (121, 222)	159 (119, 207)	1.69 (0.50, 5.78)	0.4014	0.2954
Apoptosis response ^b^	115 (80, 141)	131 (107, 159)	0.51 (0.14, 1.86)	0.3093	0.1475
B-cell regulation ^c^	197 (149, 222)	204 (160, 265)	0.65 (0.19, 2.26)	0.4979	0.4966
Cell Metabolism ^d^	87 (71, 108)	82.5 (56.5, 105)	3.88 (0.97, 15.56)	0.554	0.0265
Chemotaxis/Activation/recruitment ^e^	540 (381, 675)	513 (344, 586)	1.96 (0.55, 7.03)	0.3009	0.1103
Cytotoxicity ^f^	99 (69, 146)	122 (96, 152)	0.66 (0.18, 2.39)	0.5235	0.4338
T-cell differentiation ^g^	201 (167, 259)	187 (138, 254)	1.89 (0.54, 6.69)	0.3222	0.3733
Growth factors ^h^	411 (259, 467)	421 (317, 521)	0.62 (0.17, 2.27)	0.4686	0.7755
Inflammation ^i^	319 (248, 370)	329 (264, 404)	0.53 (0.13, 2.10)	0.3660	0.4458
Interferons ^l^	87.5 (55, 140)	130 (99.5, 183)	0.16 (0.03, 0.7)	0.0155	0.0281
Morphogenetic factors ^j^	156 (138, 213)	164 (126, 183)	3.03 (0.84, 10.97)	0.0913	0.2754
TH2 regulation ^k^	72.5 (50, 150)	127 (85, 197)	0.24 (0.06, 0.96)	0.0439	0.0563

^a^IL-10, IL-19, IL-1RA, IL-6RA; ^b^FASL, FAS, PD-L1; ^c^APRIL, BAFF, CD40, IL-5, TACI; ^d^lep, lep-R; ^e^CCL11, CCL19, CCL20, CCL2, CCL3, CCL4, CCL5, CX3CL1, CXCL10, CXCL1, CXCL2, OPN; ^f^GRZB, TNF, TRAIL; ^g^IL-12p70, IL-15, IL-27, IL-2, IL-7; ^h^EGF, FGF-basic, FLT-3 ligand, GM-CSF, G-CSF, IL-11, IL-3, PDGF-AA, PDGF-AB, VEGF; ^i^IL-17C, IL-17E, IL-17, IL-18, IL-1B, IL-1A, IL-23, IL-6; ^l^IFNα, IFNβ, IFNγ; ^j^BMP-2, BMP-4, BMP-7, TGFα; ^k^IL-13, IL-33, IL-4;

^#^ Mechanical ventilation or death; ^$^ p-value for highest tertile vs lowest tertile of ranks from a logistic regression model including age, gender and age-unadjusted CCI. ^£^p-value for rank sum as continuous variable, as above.

Considering that when analysis performed by Mann-Whitney test and logistic regression, FAS, FAS-L, IL-5, Lep-R, CCL11, CCL20, CXCL1, CXCL10, CX3CL1, OPN, TRAIL, IL-27, IL-2, EGF, PDGF-AB/BB, IL-1α, IL-17, IL-18, IFNβ, IFNγ, and IL-13 were significantly different with both tests, and that Lep-R, IFNβ, IFNγ and IL-13 are collected in the group related to interferons and TH2 regulation which are predictive of life-threatening COVID-19, we could conclude that the levels of these molecules are the most important factors, at least in our cohort, in determining the risk for mechanical ventilation and/or death in our cohort of patients with COVID-19.

## Discussion

Our analysis, based on the fine and simultaneous quantification of 62 soluble molecules, suggests that besides known risk factors, such as age and comorbidities, altered levels of several cytokines, chemokines were also independently associated with the risk of IMV or in-hospital death. A central question in COVID-19 is to figure out how SARS-CoV-2 elicits heterogeneity in disease severity and immunopathology. Here, we aimed at deciphering, at least in part, the prognostic role of several cytokines and chemokines to predict a clinical outcome. Besides examining the association with each molecule individually, because of the relatively small sample size and limited number of participants with an unfavourable outcome, we also took a more comprehensive approach by stratifying molecules according to a pre-specified functional grouping (17).

We found that soluble lep-R was increased in patients who received mechanical ventilation or died. This receptor has the main lectin-binding activity in human blood, forming complexes with free leptin and preventing its degradation (19). Although lep-R could represent a potential reservoir of bioactive lep, it could also suppress leptin action through inhibition of its binding to the membrane-bound receptor (19). As a result, a full comprehension of its function in physiological and pathological is still missing. Lep is secreted by adipocytes, and acts as a hormone regulating appetite and energy (20). It shares structural homology with IL-6, IL-11, IL-12 and oncostatin M, and stimulates the proliferation of several types of immune cells, including monocytes, natural killer cells and T helper cells. For these reasons it can be considered a proinflammatory cytokine (20). Previous reports indicate that lep dysregulation is linked to cytokine storm in COVID-19, and that in obese patients with COVID-19 the interplay between lep and inflammatory cytokines is linked with high morbidity and mortality (21). Here, we show that lep-R is elevated in patients who received mechanical ventilation or who died. In this setting, the lep-R could trigger a mechanism that counteracts inflammation and prevents the proliferation of inflammatory cells.

Cytokines coordinating TH2 response, including IL-13, were also modified in patients with clinical deterioration. Previous reports on immune response to SARS-CoV-2 have shown that T cell response is greatly diverse, as T cells from COVID-19 patients can secrete TH1 cytokines, including IFN-γ and TNF, TH17 cytokines, including IL-17A, TH2 cytokines, including IL-4, and others, including IL-2 and CD107a (9, 22). This heterogeneity makes it demanding to find specific indicators of disease and to select possible therapies. In this study, we found that IL-13 is associated with severe outcomes. In line with this, using a mouse model of COVID-19, it has been shown that IL-13 promotes severe disease, and that this response is likely to be mediated by the deposition of hyaluronan in lungs (23). TH2 cytokines, in particular IL-4 and IL-13, have also a role in the differentiation of M2 macrophages, that in turn have a role in the development of pulmonary fibrosis through the secretion of TGF-β (24). Higher expression of TGF-β, IL-13 and IL-4 has been described also in post-mortem lung biopsies obtained from two patients who died for COVID-19 (25). Few data are available on the quantification of IL-13 in plasma from COVID-19 patients. We found that IL-13 was reduced in plasma from patients who received mechanical ventilation or who died. IL-13 positively regulates the profibrotic actions of TGF-β. Unfortunately, in our study we could not measure TGF-β1, -β2, -β3 due to lack of biological material required to activate samples for their quantification.

The functional grouping including IFN-γ was also associated with the higher risk of a worse clinical outcome. Type I IFNs have an important role during viral infections and in the immunobiology of COVID-19 (26, 27). Indeed, loss-of-function mutations in genes involved in type I IFNs pathways have been described in a proportion of patients with severe COVID-19 (2). In addition, autoantibodies with neutralizing capacity against type I IFNs have been detected in patients with life-threatening COVID-19 (28). Here, we found that IFN-β and IFN-γ were decreased in COVID-19 patients who underwent mechanical ventilation or who died when compared to discharged patients. High levels of IFN-γ, which is crucial for T and NK cell activation, were detected in patients with clinical deterioration. This is in agreement with previous data showing that higher levels of IFN-γ were related to a poorer prognosis (29).

We observed that chemokines, *i.e.* crucial biomarkers needed during immune responses for chemotaxis, activation and recruitment of immune cells, were altered at hospital admission in patients who eventually required intubation or died for the disease. During SARS-CoV-2 infection, the virus life cycle causes the release of a variety of inflammatory molecules such as those containing damage associate molecular patterns (DAMPs) from the host cells. DAMPs include ATP, oligomers and nucleic acids which induce lung epithelial cells, endothelial cells and alveolar macrophages to secrete cytokines and chemokines to recruit monocytes, macrophages and T cells which in turn release IFN-γ and other pro-inflammatory cytokines that further boost inflammation, so generating an activatory loop which end up in lung injury (30). Elevated levels of these molecules in the circulation could be responsible, at least in part, for multiple organ damage in fatal COVID-19 (31). Among these chemokines, CXCL10 is of particular interest because of its high plasma level, even in the presence of lower IFN-γ levels, and for its well-known association with increased viral load, lung deterioration and a fatal outcome (32, 33). Our data show that higher levels of CXCL10 are associated with a 2.2-fold increase risk of unfavourable clinical outcome in our sample. CXCL10, together with CXCL9 and CXCL11, is the ligand of CXCR3 receptor (34), whose expression on immune cells is relevant for homing to the lung (35). Interestingly, in acute respiratory distress (ARDS) models, CXCL10 and/or CXCR3 knock-out mice showed decreased lung injury severity and increased survival in response to both viral and non-viral lung injury (36). Our analysis also shows that elevated levels of CCL2 were present in patients who underwent IMV or who died for the disease. This result is consistent with those of other studies showing increased levels of CCL2 in patients with COVID-19 pneumonia (9). CCL2 is known for its ability to regulate the chemotaxis of both myeloid and lymphoid cells, in physiological and pathological conditions (37). However, emerging evidences suggest that the functions of CCL2 could be expanded beyond its original characterization as a chemoattractant. Indeed, data show that it can drive leukocyte behavior, influencing adhesion, polarization, effector molecule secretion, autophagy, killing, and survival. Therefore, its involvement in COVID-19 deserves further studies and renders it an interesting therapeutic target (37).

One limitation of our study is the lack of non-COVID-19-related ARDS disease groups that would facilitate direct comparison between different infections. However, since few cases of these diseases have been reported during COVID-19 pandemic, it would be extremely difficult to find a similar number of patients comparable to that of COVID-19. Secondly, quantifications were performed at a single time-point, *i.e.* at hospital admission, and were not performed over time, namely during recovery. This could limit interpretations for clinical implementation. Further, all analyses were controlled only for age, gender and extent of comorbidities, so we cannot rule out bias due to other confounding factors. In any case, to our knowledge, this is one of the first studies in COVID-19 disease which uses a global, non-parametric approach to analysis.

In conclusion, we have identified a number of plasma biomarkers and their combination that change in patients with clinical deterioration and that, if confirmed in other cohorts, may help identifying patients with life-threatening COVID-19. It is now clear that the action of a single mediator of the immune system (a given cytokine, chemokine, or a soluble factor) is not sufficient to fully unfold the immunopathogenesis of fatal COVID-19. In fact, we believe that the interplay of different components could be of paramount importance for driving the final detrimental effect. However, further studies on larger groups of patients are needed to better clarify the exact prognostic value of these biomarkers.

## Data Availability Statement

Raw data supporting the conclusions of this article are available upon request.

## Ethics Statement

The studies involving human participants were reviewed and approved by Comitato Etico dell’Area Vasta Emilia Nord, protocol number 177/2020, March 11th, 2020. The patients/participants provided their written informed consent to participate in this study.

## Author Contributions

CM, AC-L, AC: study conception and design. LGi, SBD, AC-L: data analysis and interpretation; drafting the manuscript. LGo, MMa, DLT, AP, RB, LF, AN: acquisition of data. MMe, SB, MG, GG, CM: patients’ recruitment. All authors contributed to the article and approved the submitted version.

## Funding

This study was supported by Ministero della Salute, Bando Ricerca COVID-19 (2020–2021) to AC, grant number: COVID-2020-12371808. The authors declare that this study also received funding from Glem Gas SpA (San Cesario, MO, Italy), Sanfelice 1893 Banca Popolare (San Felice sul Panaro, MO, Italy), C.O.F.I.M SPA & Gianni Gibellini, Franco Appari, Assicuratrice Milanese, Andrea Lucchi, Federica Vagnarelli, Biogas Europa Service & Massimo Faccia, Alberto Bertoli, Maria Santoro, Valentina Spezzani, Gruppo BPER, Rotary Club Distretto 2072 (Clubs in Modena, Modena L.A. Muratori, Carpi, Sassuolo, Castelvetro di Modena) and Pierangelo Bertoli Fans Club. These funders were not involved in the study design, collection, analysis, interpretation of data, the writing of this article or the decision to submit it for publication.

## Conflict of Interest

The authors declare that the research was conducted in the absence of any commercial or financial relationships that could be construed as a potential conflict of interest.

## Publisher’s Note

All claims expressed in this article are solely those of the authors and do not necessarily represent those of their affiliated organizations, or those of the publisher, the editors and the reviewers. Any product that may be evaluated in this article, or claim that may be made by its manufacturer, is not guaranteed or endorsed by the publisher.

## References

[1] Morens DM Fauci AS. Emerging Pandemic Diseases: How We Got to COVID-19. Cell (2020) 183(3):837. doi: 10.1016/j.cell.2020.08.021 33125895 PMC7598893

[2] Zhang Q Bastard P Liu Z Le Pen J Moncada-Velez M Chen J. Inborn Errors of Type I IFN Immunity in Patients With Life-Threatening COVID-19. Science (2020) 370(6515):eabd4570. doi: 10.1126/science.abd4570 32972995 PMC7857407

[3] Merad M Martin JC. Pathological Inflammation in Patients With COVID-19: A Key Role for Monocytes and Macrophages. Nat Rev Immunol (2020) 20(6):355–62. doi: 10.1038/s41577-020-0331-4 PMC7201395 32376901

[4] Thwaites RS Sanchez Sevilla Uruchurtu A Siggins MK Liew F Russell CD Moore SC. Inflammatory Profiles Across the Spectrum of Disease Reveal a Distinct Role for GM-CSF in Severe COVID-19. Sci Immunol (2021) 6(57):eabg9873. doi: 10.1126/sciimmunol.abg9873 33692097 PMC8128298

[5] Vanderbeke L Van Mol P Van Herck Y De Smet F Humblet-Baron S Martinod K. Monocyte-Driven Atypical Cytokine Storm and Aberrant Neutrophil Activation as Key Mediators of COVID-19 Disease Severity. Nat Commun (2021) 12(1):4117. doi: 10.1038/s41467-021-24360-w 34226537 PMC8257697

[6] Iwamura APD Tavares da Silva MR Hummelgen AL Soeiro Pereira PV Falcai A Grumach AS. Immunity and Inflammatory Biomarkers in COVID-19: A Systematic Review. Rev Med Virol (2021) 31(4):e2199. doi: 10.1002/rmv.2199 34260778

[7] De Biasi S Tartaro DL Gibellini L Paolini A Quong A Petes C. Endogenous Control of Inflammation Characterizes Pregnant Women With Asymptomatic or Paucisymptomatic SARS-CoV-2 Infection. Nat Commun (2021) 12(1):4677. doi: 10.21203/rs.3.rs-263619/v1 34326336 PMC8322155

[8] De Biasi S Lo Tartaro D Meschiari M Gibellini L Bellinazzi C Borella R. Expansion of Plasmablasts and Loss of Memory B Cells in Peripheral Blood From COVID-19 Patients With Pneumonia. Eur J Immunol (2020) 50(9):1283–94. doi: 10.1002/eji.202048838 32910469

[9] De Biasi S Meschiari M Gibellini L Bellinazzi C Borella R Fidanza L. Marked T Cell Activation, Senescence, Exhaustion and Skewing Towards TH17 in Patients With COVID-19 Pneumonia. Nat Commun (2020) 11(1):3434. doi: 10.21203/rs.3.rs-23957/v1 32632085 PMC7338513

[10] Gibellini L De Biasi S Paolini A Borella R Boraldi F Mattioli M. Altered Bioenergetics and Mitochondrial Dysfunction of Monocytes in Patients With COVID-19 Pneumonia. EMBO Mol Med (2020) 12(12):e13001. doi: 10.15252/emmm.202013001 33078545 PMC7645870

[11] Cossarizza A De Biasi S Guaraldi G Girardis M Mussini C Modena Covid-19 Working G. SARS-CoV-2, the Virus That Causes COVID-19: Cytometry and the New Challenge for Global Health. Cytometry A (2020) 97(4):340–3. doi: 10.1002/cyto.a.24002 PMC7162395 32187834

[12] Cossarizza A Gibellini L De Biasi S Lo Tartaro D Mattioli M Paolini A. Handling and Processing of Blood Specimens From Patients With COVID-19 for Safe Studies on Cell Phenotype and Cytokine Storm. Cytometry A (2020) 97(7):668–73. doi: 10.1002/cyto.a.24009 PMC7262259 32275124

[13] Fajgenbaum DC June CH. Cytokine Storm. N Engl J Med (2020) 383(23):2255–73. doi: 10.1056/NEJMra2026131 PMC7727315 33264547

[14] Paolini A Borella R De Biasi S Neroni A Mattioli M Lo Tartaro D. Cell Death in Coronavirus Infections: Uncovering Its Role During COVID-19. Cells (2021) 10(7):1585. doi: 10.3390/cells10071585 34201847 PMC8306954

[15] Rebecca Borella SDB Paolini A Boraldi F Lo Tartaro D Mattioli M Guaraldi G. Metabolic Reprograming Shapes Neutrophil Functions in Severe COVID-19. Eur J Immunol (2021) 52:484–502. doi: 10.1002/EJI.202149481/v3/response1 34870329

[16] Gil-Etayo FJ Suarez-Fernandez P Cabrera-Marante O Arroyo D Garcinuno S Naranjo L. T-Helper Cell Subset Response Is a Determining Factor in COVID-19 Progression. Front Cell Infect Microbiol (2021) 11:624483. doi: 10.3389/fcimb.2021.624483 33718270 PMC7952877

[17] Cossarizza A Chang HD Radbruch A Abrignani S Addo R Akdis M. Guidelines for the Use of Flow Cytometry and Cell Sorting in Immunological Studies (Third Edition). Eur J Immunol (2021) 51(12):2708–3145. doi: 10.1002/eji.202170126 34910301 PMC11115438

[18] Davey RT Jr. Lynfield R Dwyer DE Losso MH Cozzi-Lepri A Wentworth D. The Association Between Serum Biomarkers and Disease Outcome in Influenza A(H1N1)pdm09 Virus Infection: Results of Two International Observational Cohort Studies. PLoS One (2013) 8(2):e57121. doi: 10.1371/journal.pone.0057121 23468921 PMC3584122

[19] Zastrow O Seidel B Kiess W Thiery J Keller E Bottner A. The Soluble Leptin Receptor is Crucial for Leptin Action: Evidence From Clinical and Experimental Data. Int J Obes Relat Metab Disord (2003) 27(12):1472–8. doi: 10.1038/sj.ijo.0802432 14634677

[20] La Cava A Matarese G. The Weight of Leptin in Immunity. Nat Rev Immunol (2004) 4(5):371–9. doi: 10.1038/nri1350 15122202

[21] Maurya R Sebastian P Namdeo M Devender M Gertler A. COVID-19 Severity in Obesity: Leptin and Inflammatory Cytokine Interplay in the Link Between High Morbidity and Mortality. Front Immunol (2021) 12:649359. doi: 10.3389/fimmu.2021.649359 34220807 PMC8250137

[22] Weiskopf D Schmitz KS Raadsen MP Grifoni A Okba NMA Endeman H. Phenotype and Kinetics of SARS-CoV-2-Specific T Cells in COVID-19 Patients With Acute Respiratory Distress Syndrome. Sci Immunol (2020) 5(48):eabd2071. doi: 10.1126/sciimmunol.abd2071 32591408 PMC7319493

[23] Donlan AN Sutherland TE Marie C Preissner S Bradley BT Carpenter RM. IL-13 is a Driver of COVID-19 Severity. medRxiv (2021). doi: 10.1172/jci.insight.150107 PMC8410056 34185704

[24] Vaz de Paula CB de Azevedo MLV Nagashima S Martins APC Malaquias MAS Miggiolaro A. IL-4/IL-13 Remodeling Pathway of COVID-19 Lung Injury. Sci Rep (2020) 10(1):18689. doi: 10.1038/s41598-020-75659-5 33122784 PMC7596721

[25] Ribeiro Dos Santos Miggiolaro AF da Silva Motta Junior J Busatta Vaz de Paula C Nagashima S Alessandra Scaranello Malaquias M Baena Carstens L. Covid-19 Cytokine Storm in Pulmonary Tissue: Anatomopathological and Immunohistochemical Findings. Respir Med Case Rep (2020) 31:101292. doi: 10.1016/j.rmcr.2020.101292 33200067 PMC7658564

[26] Alavi Darazam I Shokouhi S Pourhoseingholi MA Naghibi Irvani SS Mokhtari M Shabani M. Role of Interferon Therapy in Severe COVID-19: The COVIFERON Randomized Controlled Trial. Sci Rep (2021) 11(1):8059. doi: 10.21203/rs.3.rs-136499/v1 33850184 PMC8044200

[27] Pairo-Castineira E Clohisey S Klaric L Bretherick AD Rawlik K Pasko D. Genetic Mechanisms of Critical Illness in COVID-19. Nature (2021) 591(7848):92–8. doi: 10.1038/s41586-020-03065-y 33307546

[28] Bastard P Rosen LB Zhang Q Michailidis E Hoffmann HH Zhang Y. Autoantibodies Against Type I IFNs in Patients With Life-Threatening COVID-19. Science (2020) 370(6515):eabd4585. doi: 10.1126/science.abd4585 32972996 PMC7857397

[29] Gadotti AC de Castro Deus M Telles JP Wind R Goes M Garcia Charello Ossoski R. IFN-Gamma is an Independent Risk Factor Associated With Mortality in Patients With Moderate and Severe COVID-19 Infection. Virus Res (2020) 289:198171. doi: 10.1016/j.virusres.2020.198171 32979474 PMC7510544

[30] Coperchini F Chiovato L Rotondi M. Interleukin-6, CXCL10 and Infiltrating Macrophages in COVID-19-Related Cytokine Storm: Not One for All But All for One! Front Immunol (2021) 12:668507. doi: 10.3389/fimmu.2021.668507 33981314 PMC8107352

[31] Coperchini F Chiovato L Croce L Magri F Rotondi M. The Cytokine Storm in COVID-19: An Overview of the Involvement of the Chemokine/Chemokine-Receptor System. Cytokine Growth Factor Rev (2020) 53:25–32. doi: 10.1016/j.cytogfr.2020.05.003 32446778 PMC7211650

[32] Osuchowski MF Winkler MS Skirecki T Cajander S Shankar-Hari M Lachmann G. The COVID-19 Puzzle: Deciphering Pathophysiology and Phenotypes of a New Disease Entity. Lancet Respir Med (2021) 9(6):622–42. doi: 10.1016/S2213-2600(21)00218-6 PMC8102044 33965003

[33] Yang Y Shen C Li J Yuan J Wei J Huang F. Plasma IP-10 and MCP-3 Levels are Highly Associated With Disease Severity and Predict the Progression of COVID-19. J Allergy Clin Immunol (2020) 146(1):119–127 e114. doi: 10.1016/j.jaci.2020.04.027 32360286 PMC7189843

[34] Rotondi M Chiovato L Romagnani S Serio M Romagnani P. Role of Chemokines in Endocrine Autoimmune Diseases. Endocr Rev (2007) 28(5):492–520. doi: 10.1210/er.2006-0044 17475924

[35] Alon R Sportiello M Kozlovski S Kumar A Reilly EC Zarbock A. Leukocyte Trafficking to the Lungs and Beyond: Lessons From Influenza for COVID-19. Nat Rev Immunol (2021) 21(1):49–64. doi: 10.1038/s41577-020-00470-2 33214719 PMC7675406

[36] Ichikawa A Kuba K Morita M Chida S Tezuka H Hara H. CXCL10-CXCR3 Enhances the Development of Neutrophil-Mediated Fulminant Lung Injury of Viral and Nonviral Origin. Am J Respir Crit Care Med (2013) 187(1):65–77. doi: 10.1164/rccm.201203-0508OC 23144331 PMC3927876

[37] Gschwandtner M Derler R Midwood KS. More Than Just Attractive: How CCL2 Influences Myeloid Cell Behavior Beyond Chemotaxis. Front Immunol (2019) 10:2759. doi: 10.3389/fimmu.2019.02759 31921102 PMC6923224

